# Applying theory of planned behavior to examine adolescent female athletes’ intentions of continued sport participation

**DOI:** 10.3389/fspor.2026.1794861

**Published:** 2026-06-16

**Authors:** Chris Hopkins, Casey S. Hopkins

**Affiliations:** 1 Department of Health Sciences, Furman University, Greenville, SC, United States; 2 School of Nursing, Clemson University, Clemson, SC, United States

**Keywords:** adolescent female, female athlete, physical activity, sport participation, structural equation modeling, theory of planned behavior

## Abstract

**Introduction:**

Adolescent females discontinue sports at higher rates than males, which contributes to their lower overall physical activity during this important period of development. Potentially useful for understanding this disparity, the Theory of Planned Behavior (TPB) provides a framework to study one's intentions through their attitudes, subjective norms, and perceived behavioral control. This study examined whether TPB constructs predict female adolescents’ intentions to continue participating in sports and remain physically active.

**Methods:**

This cross-sectional study used an online questionnaire to collect data from 271 female adolescents who recently participated in organized sports. Structural equation modeling was used to test TPB's ability to predict participants’ intentions of continued sports participation and their intentions of regular exercise in one year.

**Results:**

Structural equation models for both outcomes exhibited acceptable fit. Participants’ intention of participating in sports next year was predicted by attitudes (*β* = 0.84, *p* < 0.001) and subjective norms (*β* = 0.35, *p* = 0.005). Participants’ intentions of exercising next year were predicted by attitudes (*β* = 0.30, *p* = 0.009), subjective norms (*β* = 0.27, *p* = 0.009), and perceived behavioral control (*β* = 0.28, *p* = 0.001). These findings support TPB as a useful framework for understanding female adolescents’ intentions and highlight the importance of enjoyment, parental support, and peer acceptance in sustaining sports participation and physical activity.

**Discussion:**

Findings support TPB as a useful framework for understanding adolescent female athletes’ intentions of continued sports participation and future physical activity. Based on these findings, interventions to enhance continued girls’ sports participation should promote supportive, confidence-building environments, foster encouraging social interactions between teammates and peers, and reduce structural barriers related to access and cost. These approaches align with public health priorities and support pathways to lifelong engagement in sport and physical activity.

## Introduction

Physical activity is a key component in maintaining adequate health. The World Health Organization (WHO) recommends youth aged 5–17 years should participate in at least 60 min of moderate-to-vigorous physical activity each day, but globally, 81% of adolescents aged 11–17 years did not meet these guidelines in 2020 (1, 2). Adolescent girls compared to their male counterparts tend to be less physically active (3). Participation in sports is a common method adolescents can use to meet their physical activity needs. Beyond physical benefits, sport involvement has been linked to improved academic, social, and economic outcomes (4–11). Adolescents taking part in sports have demonstrated greater levels of self-esteem and decreased risk for depression (7, 12, 13). Behaviors related to mental health and the decreased depression risk including improved dietary intake, safer sexual practices, and lower rates of substance abuse have also been linked to youth sport participation (13). Despite the vast benefits of physical activity through sport participation, children have been found to begin dropping out of their sport as early as 8 years old.13 Girls tend to begin playing sports later than boys, participate at a lower level across grades 3–12 (age 8–17 years), and drop out of sports at two times the rate of boys by age 14 (14).

Recognizing the importance of childhood physical activity, Healthy People 2030 includes an objective (PA-12) to increase the proportion of children and adolescents who play sports (4). The objective was set with a baseline of 58.3% of American children and adolescents aged 6–17 years having participated in a sports team or taken after school sports lessons within the past 12 months in 2016–2017 reports. Unfortunately, since then the status has worsened with 54.6% involved in sports in 2022–2023. Over time, the gap in participation between boys and girls has increased, reflecting a disproportionate decline in participation among girls, from a 6.3 percentage-point difference in 2016–2017 to a 9.3 percentage-point difference in 2022–2023 (4).

Reasons for gender disparities are multifaceted. Some factors impacting girls’ sport participation include biological development, social stigma against females in sport, gender stereotypes, parental support, lack of positive role modeling, safety and transportation issues, and other socioeconomic issues (14). Pubertal development may impact girls’ decision to continue with sport participation (15). In a 3-year longitudinal study, there was significantly reduced participation in organized individual sport during late pubertal stages among girls when compared to boys. The same association was not true for organized team sports, which researchers suggested could be attributed to empathy and support for shared experiences that girls could share with their female teammates (15). In a large systematic review of menstrual status and physical activity, menarche and subsequent periods, which may cause physical discomfort and/or social anxiety related to bleeding, were found to negatively impact girls’ motivation and willingness to participate in physical activities (16).

Aside from biological factors, the Women’s Sports Foundation has outlined other important barriers to female sport participation, including family finances, lack of parental engagement, opportunities to participate, injury or health concerns, poor coaching, and lack of women role models (17). The physical environment available for sport participation, along with perception of safety, is also a common barrier (18). These barriers are substantiated by recent systematic reviews (19–21).

While there are robust scientific findings elucidating factors that influence sport participation and drop out, the translation of those findings into real-world applications to improve outcomes is limited. Researchers have called for studies based on theory to explicate the complexity of factors influencing physical activity among girls (21). In a systematic review of factors associated with sport participation among adolescent females, the majority of studies in this field did not use a theoretical framework (20). The application of theory in research can inform the development and design of practical applications for implementing policies and practice changes (22). Therefore, the purpose of this study was to identify factors contributing to adolescent girls’ intentions for continued sport participation and future physical activity guided by the Theory of Planned Behavior in an effort to explain relationships between contributing factors and intentions and to create pathways to practical strategies to improve health outcomes within this population.

## Methods

### Study design

This cross-sectional study used an online questionnaire to examine determinants of adolescent girls’ intentions of continued sports participation and future physical activity, guided by the Theory of Planned Behavior. Data were collected from adolescent girls in South Carolina who participated in at least one organized sport during the previous year. The questionnaire included items related to participant demographics, TPB constructs, and participants’ intentions of continued sports participation and future physical activity. The questionnaire was distributed with the assistance of high school administrators and coaches throughout South Carolina, who then shared a link to the online questionnaire with eligible participants. The study was designed to enable efficient and simultaneous assessment of hypothesized TPB constructs and participants’ intentions of continued sports participation and future physical activity. Institutional Review Board approval for this study was granted by Clemson University.

### Theoretical framework

The Theory of Planned Behavior (TPB) is a social cognitive framework commonly used to understand motivation and behaviors in many contexts, including exercise and physical activity (23). The TPB expands upon the Theory of Reasoned Action to include behaviors in which individuals have incomplete volitional control, or in other words, behaviors that an individual can decide to perform or not perform, given they have the required opportunities and resources (24). Structurally, the TPB consists of three constructs: attitudes, subjective norms, and perceived behavioral control (24). Attitudes refer to how much an individual has either a positive or adverse assessment of the behavior, subjective norms deal with the social pressure that is faced to act upon the behavior or not, and perceived behavioral control relates to the perceived ease or difficulty of performing the behavior (24).

### Questionnaire

The questionnaire was comprised of 77 items, including three items related to age, grade level, and ethnicity; six items assessing family affluence using the Family Affluence Scale (FAS-III), validated for use with adolescent research participants (25); three items related to participants’ sports participation including which sport they consider their primary sport and their duration of participation; two items related to their history of injuries while participating in sports; twelve items adapted from the parental support for physical activity scale (26); seven items from the Coach-Athlete Relationship Questionnaire (27); four items assessing enjoyment in sports participation from the Sport Commitment Model (28); ten items from the Athletic and Social subscales of the Self-Perception Profile for Adolescents (29); twenty-eight items from the Sport Motivation Scale (30); and two original items measuring participants’ intentions of sports participation and physical activity in one year. Before distribution, the study team reviewed all questionnaire items for clarity, relevance, and appropriateness for adolescent female athletes.

### Outcome measures

The two dependent variables in this study were participants’ intentions of future sport participation and their intentions of regular future exercise. Intentions of future sport participation was assessed with the following prompt, “On a scale from 0 to 100, with 100 being the most confident, how confident are you that you will continue playing your primary sport next year?” Intentions of regular future exercise was assessed with the following prompt, “On a scale from 0 to 100, with 100 being the most confident, how confident are you that you will exercise most days of the week next year?”

### Theory of planned behavior constructs

Construct validity of the Theory of Planned Behavior measures was assessed using confirmatory factor analysis (CFA). Scales were considered an acceptable model fit if Likelihood ratio Chi-squared tests (χχ2) failed to reject the null hypothesis, Bentler's (31) comparative fit indexes (CFI) were above.90, Steiger & Lind's (32) root-mean-square error of approximation (RMSEA) was less than.08, or the root-mean-square residual (SRMR) was less than.08. Internal consistency reliability for each scale was evaluated using Cronbach's alpha with *α*>0.70 considered acceptable reliability for this study. Means (M) and standard deviations (SD) are also included for each of the scales below.

A latent variable for attitudes was formed using eight items, including one item from the Sport Commitment Model (28) regarding participants’ enjoyment of playing their primary sport this year, five items measuring intrinsic motivation from the Sport Motivation Scale (30), and three items measuring extrinsic motivation from the Sport Motivation Scale (30). CFA indicated that the items formed an acceptable latent variable representing attitudes, χχ2(20) = 30.84, *p* = 0.06, CFI = 0.98, RMSEA = .05, SRMR = .04. Internal consistency reliability for the eight-item scale was acceptable (*α* = 0.74, M = 0.78, SD = 0.12).

A latent variable for subjective norms was formed using eight items, including four items from the Social Subscale of the Self-Perception Profile for Adolescents (29) and four items adapted from the parental support for physical activity scale (26). CFA indicated that the items formed an acceptable latent variable representing subjective norms, χχ2(5) = 9.38, *p* = 0.09, CFI = 0.98, RMSEA = .06, SRMR = .03. Internal consistency reliability for the eight-item scale was acceptable (*α* = 0.74, M = 0.60, SD = 0.13).

A latent variable for perceived behavioral control was assessed with four items from the athletic subscale of the Self-Perception Profile for Adolescents (29). CFA indicated that the items formed an acceptable latent variable representing perceived athletic competence, χχ2(2) = 5.92, *p* = 0.052, CFI = 0.99, RMSEA = .08, SRMR = .03. Internal consistency reliability for the four-item scale was acceptable (*α* = 0.81, M = 0.71, SD = 0.17).

Two additional variables, coach-athlete relationship quality and family affluence, were considered key background factors important to the TPB. The quality of the coach-athlete relationship was selected as an antecedent of attitudes, as it can shape an athlete's affective evaluations, enjoyment, and perceived value of sport and exercise participation (33–35). Family affluence was selected as an antecedent of subjective norm, as a family's socioeconomic resources can shape the way a youth athlete normalizes physical activity and sports participation (20, 36, 37). These antecedent paths are considered as contextual variables specified to predict attitudes and subjective norm, but not directly predicting intentions. Coach-athlete relationship quality measured with the seven items forming the closeness and commitment dimensions of the Coach-Athlete Relationship Questionnaire (27). Family affluence was measured with the Family Affluence Scale (FAS-III) (25). The FAS-III has been validated in many countries and used to measure associations between family affluence and various health outcomes among adolescents (25).

### Statistical analysis

Descriptive statistics were analyzed for the sample, including age, ethnicity, family affluence, primary sport, and the dependent variables measuring participants’ likelihood of playing their primary sport and exercising most days next year. For easier interpretation as a descriptive statistic, family affluence was categorized into low affluence, medium affluence, and high affluence using methods in a previous study (38). For all descriptive statistics reported, means and standard deviations were used to describe continuous variables and frequencies with sample proportions were used to describe categorical variables.

Intercorrelations were calculated for all TPB constructs, future sport participation intentions, and future exercise intentions. Maximum Likelihood structural equation modeling was used to test the TPB framework in relation to intentions of future sports and exercise participation. Consistent with TPB, attitudes, subjective norms, and perceived behavioral control were specified as simultaneous predictors of sport and exercise intentions. Also consistent with TPB, background variables were identified as predictors of key TPB constructs rather than direct predictors of sports and exercise intentions. The standards used to assess acceptable model fitness for TPB construct scales were also used to assess the hypothesized structural equation models (χχ2, CFI, RMSEA, and SRMR). Models were estimated for future sports participation and exercise intentions. All analyses were performed using Stata 18.0 statistical software (Stata Corp LP, College Station, Texas, USA).

## Results

### Descriptive statistics

 Table 1 reports characteristics for the 271 adolescent females in the study. This sample size is adequate to power the proposed structural equation model, which contains 18 parameters. This 15:1 participant-to-parameter ratio exceeds general recommendations for SEM (39, 40). Participants varied in age from 13 to 18 years old. Over three-quarters of participants reported their ethnicity as white, and most participants reported medium family affluence based on their responses to the FAS-III. Participants were more diverse in their reported primary sport, with over 17 different sports represented in the sample. Overall, participants reported a high likelihood of playing their primary sport and exercising regularly next year.

**Table 1:** Participant characteristics (*n* = 271).

Variable	Value
Age; Years (SD)	15.6 (1.59)
Ethnicity; *n* (%)	White: 206 (76.0%)
	Hispanic/Latino: 24 (8.9%)
	Black: 19 (7.0%)
	Asian: 8 (3.0%)
	Other: 14 (5.2%)
Family Affluence Scale; Mean (SD)	Low (0–7): 50 (18.5%)
	Medium (8–11): 205 (75.7%)
	High (12–13): 16 (5.9%)
Primary Sport; Sport: *n* (%)	Soccer: 75 (27.7%)
	Cross Country: 41 (15.1%)
	Volleyball: 36 (13.3%)
	Softball: 25 (9.2%)
	Lacrosse: 24 (8.9%)
	Basketball: 17 (6.3%)
	Track & Field: 16 (5.9%)
	Other: 37 (13.7%)
Likelihood of Playing Sports Next Year; Mean (SD)	79.2 (27.8)
Likelihood of Exercising Most Days Next Year; Mean (SD)	81.5 (22.5)

### Measurement model

TPB constructs of attitude, subjective norm, and perceived behavioral control were weakly (r < 0.3) but significantly intercorrelated, indicating related but distinct constructs to predict intentions (attitude and subjective norms: r = 0.163, *p* = 0.007; attitude & perceived behavioral control: r = 0.295, *p* < 0.001; subjective norms & perceived behavioral control: *r* = 0.135, *p* = 0.026).

**Figure 1:**
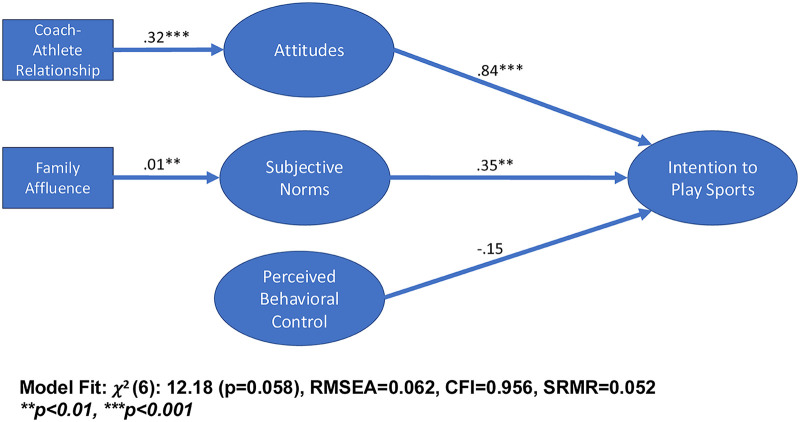
Standardized path coefficients for the theory of planned bahavior on intentions to play sports next year (*n* = 271). Model Fit: *χ*^2^(6): 12.18 (*p* = 0.058), RMSEA=0.062, CFI=0.956, SRMR=0.052. **p *<* 0.01, ***p *<* *0.001*.

 Figure 1 illustrates the model testing the TPB framework related to participants’ intentions to continue playing their primary sport next year. The overall fit of the model was acceptable [χχχχ2(6): 12.18 (*p* = 0.058), RMSEA=0.062, CFI=0.956, SRMR=0.052] and illustrated significant antecedent paths from coach-athlete relationship to attitudes (*β* = 0.32, *p* < 0.001) and family affluence to subjective norms (*β* = 0.01, *p* = 0.002). Importantly, it also estimated significant predictor paths from attitudes and subjective norms to participants’ intention to play sports next year (*β* = 0.84, *p* < 0.001 & *β* = 0.35, *p* = 0.005, respectively). Notably, this model did not include a significant predictor path from perceived behavioral control to participants’ intention to play their primary sport next year (*β* = −0.15, *p* = 0.112).

**Figure 2:**
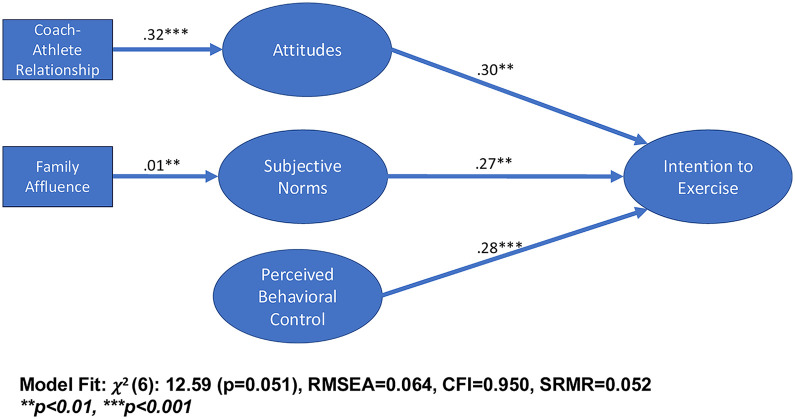
Standardized path coefficients for the theory of planned bahavior on intentions to exercise next year (*n* = 271). Model Fit: *χ*^2^(6): 12.59 (*p* = 0.051), RMSEA=0.064, CFI=0.950, SRMR=0.052. **p *<* 0.01, ***p *<* *0.001*.

 Figure 2 illustrates the model testing the TPB framework related to participants’ intentions to exercise most days next year. The overall fit of this model was acceptable [χχχχ2(6): 12.59 (*p* = 0.051), RMSEA=0.064, CFI=0.950, SRMR=0.052] and included the same significant antecedent paths from coach-athlete relationship to attitudes and family affluence to subjective norms as observed in the previous model related to sports participation. In this model, all three TPB constructs significantly predicted participants’ intentions to exercise next year. The path from attitudes to exercise intentions was the strongest (*β* = 0.30, *p* = 0.009), followed by perceived behavioral control (*β* = 0.28, *p* = 0.001), and subjective norms (*β* = 0.27, *p* = 0.009).

## Discussion

This study tested a Theory of Planned Behavior-based structural equation model to examine adolescent females’ intentions of continued sport participation and future exercise. Overall, both models exhibited acceptable fit, thus supporting the use of TPB to predict intentions. Participants’ attitudes toward sport participation served as the strongest predictor of intentions for sports participation and future exercise. Subjective norms were also an important predictor of both intentions, while perceived behavioral control effectively predicted exercise intentions, but not intentions of continued sports participation. Both models effectively incorporated interpersonal and structural antecedents to predict attitudes and subjective norms.

Interpersonally, the quality of the coach-athlete relationship was used as an antecedent, or background variable, to adolescent females’ attitudes toward sport participation. The significant path of the coach-athlete relationship to athletes’ attitudes supports the role of coaches in creating supportive interpersonal environments, which can help shape athletes’ enjoyment, satisfaction, and affective evaluation of sports experiences (34, 35, 41, 42). These affective experiences may then strengthen athletes’ intentions to continue participating in sport (20, 33, 43). Similarly, a family's affluence was used as an antecedent to adolescent females’ subjective norms toward sport participation. The path from family affluence to athletes’ subjective norms was modest (*β* = 0.01) but statistically significant. This finding suggests that socioeconomic resources play a limited, but potentially meaningful, role in shaping how female adolescents perceive social expectations around sports participation and exercise. Family resources can influence how a female adolescent perceives that her participation in sports and exercise is encouraged, supported, and normal. These perceptions of subjective norms then predict her intention to continue participating in sports.

### Core TPB constructs

Across both models for sport participation and exercise, attitudes were the strongest TPB construct to predict future intentions. Attitudes were especially strong in predicting intentions for sports participation, revealing how affectively driven sports participation is among adolescent females. This aligns well with previous research among female adolescent athletes, where continued sports participation was strongly predicted by athletes’ enjoyment of sports and their perceptions of sports participation resulting in desirable outcomes (20). Aligning with TPB, enjoyment and expected outcomes comprise female adolescents’ attitudes about participating in sports, which in turn affect their intentions of continuing participation (24).

Along with attitudes, subjective norms were also significant predictors of both sports participation and exercise intentions. This contrasts with some previous studies using TPB in exercise-related contexts (23, 44, 45), where subjective norms did not significantly predict physical activity or exercise intentions. However, those studies focused on older adults, college students, and children younger than the adolescents in the current study. Another study with adolescent females reported similar findings to the current study, with subjective norms significantly predicting physical activity among participants in a school-based physical activity program (46).

The increased importance of subjective norms among female adolescents’ sports and exercise intentions, compared to adults and children, is likely driven by the unique social expectations perceived during adolescence (47). Previous research on sports participation highlights social influence and social support as being strongly linked to adolescent sports involvement and physical activity (48–50). Adolescence is a critical time for identity development, and social interactions between female adolescent athletes and their teammates play a key role in this process (51). Additionally, parental encouragement and exercise behaviors heavily influence adolescent sports participation (20). Adolescent girls are more likely to participate in sports if their parents encourage them and regularly exercise themselves (52, 53). These strong social influences and expectations form the basis of female adolescents’ subjective norms, which significantly predict their intentions to keep participating in sports and exercising regularly.

Perceived behavioral control significantly predicted exercise intentions, but not intentions of continued sports participation. This difference may be reflective of contextual factors related to these two behaviors. When external or structural factors create barriers to behaviors, perceived behavioral control may be less influential in predicting intentions (54). Comparing organized sports participation to exercise, sports certainly have more barriers outside of an adolescent athlete's direct control. These barriers may include team selection, financial costs, time demands, and availability of organized sports opportunities (55, 56). When such outside factors exist, the influence of an adolescent's perceived behavioral control diminishes. Intentions to exercise, however, may be considered more autonomous and flexible for an adolescent to control. This may reflect an interesting association between previous sports participation and future physical activity.

Participation in organized sports tends to decrease dramatically among females from childhood through adolescence (57). Once removed from these structured, organized sports, females who participated in sports through high school are more likely to engage in regular physical activity than peers who didn’t participate in sports (58). The athletic competence they gained through years of past sports participation may enhance perceived behavioral control and drive greater intentions to exercise into adulthood.

### Practical implications

The results of this study suggest potential strategies to increase continued sports participation and exercise among adolescent girls. Attitudes exhibited the strongest influence on intentions, suggesting that interventions should prioritize athlete enjoyment, encouragement from coaches, and meaningful experiences that result in valuable skill development and social interaction. Subjective norms were also important predictors of intentions. Worthwhile interventions should encourage parental support and involvement, and foster peer interactions that normalize and value girls’ continued sports participation. Lastly, strategies targeting perceived behavioral control should reduce barriers to participation by improving access to affordable sports programs, allowing flexible scheduling, and providing opportunities to accommodate varying skill levels.

Some of these strategies may be implemented through coach education programs, where coaches learn to create supportive and confidence-building environments that build encouraging relationships between teammates and peers. Other strategies may require community support to ease financial burdens on families and reduce socioeconomic disparities in youth sport participation. Collectively, these endeavors may achieve Healthy People 2030's objective to increase child and adolescent sports participation.

### Strengths, limitations, & future research

This study has plenty of strengths, including the use of the Theory of Planned Behavior to examine two important health behaviors in a historically understudied population that faces greater problems related to sports dropout and decreased physical activity when compared to their male peers. However, limitations are also present and should be acknowledged. This cross- sectional design enabled a relatively large sample with backgrounds from diverse sports, but it did not allow for any follow-up to measure behaviors instead of intentions. Additionally, participant recruitment relied on high school administrators and coaches throughout South Carolina to help distribute the study questionnaire. This indirect method of recruitment may have limited some responses compared to more direct methods of recruitment. Lastly, the scale used for perceived behavioral control focused on general perceived athletic competence, which worked well for predicting future exercise intentions, but a sport-specific perceived competency may have been more predictive of sports participation intentions. Future studies should incorporate a longitudinal design to measure continued sports participation and exercise and consider alternative scales for perceived behavioral control related to their primary sport. Also, future studies may include interventions aiming to improve attitudes, subjective norms, and perceived behavioral control to promote greater sports continuation among adolescent females.

## Conclusion

This study reinforces the use of the Theory of Planned Behavior to examine adolescent girls’ sports and exercise intentions. Attitudes, subjective norms, and perceived behavioral control exhibited related but distinct pathways to predict intentions when also considering important antecedent factors of the coach-athlete relationship and family affluence. The significance of these antecedent factors underscores that girls’ continued sports participation is not solely their individual choice, but is influenced by important interpersonal and socioeconomic dynamics. Findings from this study suggest that strategies to enhance continued girls’ sports participation should promote supportive, confidence-building environments, foster encouraging social interactions between teammates and peers, and reduce structural barriers related to access and cost. These approaches align with public health priorities and support pathways to lifelong engagement in sport and physical activity.

## Data Availability

The original contributions presented in the study are included in the article/supplementary material, further inquiries can be directed to the corresponding author.
